# The Quality Characteristics Comparison of Stone-Milled Dried Whole Wheat Noodles, Dried Wheat Noodles, and Commercially Dried Whole Wheat Noodles

**DOI:** 10.3390/foods12010055

**Published:** 2022-12-22

**Authors:** Mengdi Cai, Chunxia Shen, Yuhui Li, Shuangli Xiong, Feng Li

**Affiliations:** 1 College of Food Science and Technology, Sichuan Tourism University, Chengdu 610100, China; 2 School of Life Science and Engineering, Southwest University of Science and Technology, Mianyang 621010, China

**Keywords:** stone milling, whole wheat noodle, microstructure, protein secondary structure, antioxidant activity, in vitro digestive property, estimated glycemic index

## Abstract

To explore the quality differences between dried wheat noodles (DWNs), stone-milled dried whole wheat noodles (SDWWNs), and commercially dried whole wheat noodles (CDWWNs), the cooking quality, texture properties, microstructure, protein secondary structure, short-range order of starch, antioxidant activity, in vitro digestive properties, and estimated glycemic index (eGI) of the noodles were investigated. The results showed that the cooking loss of SDWWNs was significantly lower than that of CDWWNs. The springiness, cohesiveness, gumminess, chewiness, and resilience of SDWWNs reached the maximum, and the tensile strength was significantly increased. The continuity of the gluten network of SDWWNs was reduced, and more holes appeared. The protein secondary structure of the SDWWNs and CDWWNs was mainly dominated by the β-sheet and β-turn, and the differences in the starch short-range order were not significant. Prior to and after the in vitro simulated digestion, the DPPH radical scavenging activity, the hydroxyl radical scavenging activity, and the total reducing power of the SDWWNs were the highest. Although the digested starch content of SDWWNs did not differ significantly from that of CDWWNs, the eGI was significantly lower than that of the CDWWNs and DWNs. Overall, the SDWWNs had certain advantages, in terms of quality characteristics.

## 1. Introduction

Wheat (*Triticum aestivum* L.) is the earliest food crop grown by humans and is grown all over the world. Whole wheat flour contains more beneficial components than refined flours, derived solely from the endosperm, such as dietary fiber, minerals, vitamins, phenolic compounds, feruloylated oligosaccharides, glutathione, octacosanol, and phytosterols [1], which has the effect of relieving mild constipation, improving the colon metabolism, and reducing the risk of obesity, metabolic syndrome, some types of cancer, type 2 diabetes, and cardiovascular diseases [2]. It is extensively used in the food processing industries, such as noodles, bread, and breakfast powder. Modern stone milling technology ameliorates the old traditional milling process, grinding, and sieving one by one, and its core superiority is the low-speed, low-temperature grinding, which avoids the high temperature caused by the continuous high-speed operation of the mechanical equipment to reduce wastage and nutrient depletion, and to ensure the maximum retention of protein, carotene, calcium, phosphorus, iron, thiamine, riboflavin, and other components [3]. The stone-milling process has the advantage of ease of use and the simplicity of system in the production of whole wheat flour. The extraction rate of stone-milled whole wheat flour is 100%, and the endosperm, germ, and bran are present in the same ratio as in the native whole grain. Pagani et al. [4] reported that stone-milled whole wheat flour has a more uniform particle size distribution than roller-milled whole wheat flour. Stone milling has little effect on the loss of the macronutrients Na, Mg, K, Ca, and P, in flour and no effect on the loss of the trace elements Mn, Fe, Cu, Zn, and Se [5]. However, stone milling generates considerable heat due to friction, compression, and shear phenomena, that might influence the nutritional and processing properties of the flour [3,6]

Processed with whole wheat flour, water, and salt as the basic ingredients, whole wheat noodles are gaining popularity among customers, due to their nutritional value, unique flavor, and ease of consumption. However, bran interferes with the formation of the gluten protein network, resulting in whole wheat noodles with an easy breakage, hard texture, poor color, and rough mouthfeel [7]. In addition, the storage stability of whole wheat noodles is easily affected by unsaturated fatty acids, lipase, lipoxygenase, and polyphenol oxidase, fatty rancidity, and enzymatic browning seriously affect its sensory quality and functional properties [8]. The current research primarily focuses on the quality characteristics of whole wheat noodles prepared by the bran re-addition method, including nutritional characteristics, sensory properties, color [9,10], cooking quality, texture properties [7,11], microstructure [12], storage stability [8,12], and volatile compounds [8], while little research has been reported on stone-milled dried whole wheat noodles. In this study, stone-milled whole wheat flour was used to prepare whole wheat noodles. The cooking quality, texture properties, microstructure, protein secondary structure, short-range order of starch, antioxidant activity, in vitro digestive properties, and estimated glycemic index (eGI) of the stone-milled dried whole wheat noodles were investigated, and the quality differences between dried wheat noodles (DWNs), stone-milled dried whole wheat noodles (SDWWNs), and commercially dried whole wheat noodles (CDWWNs) were explored, in order to provide some theoretical and application references for the development of the stone-milled flour industry and whole wheat food processing.

## 2. Materials and Methods

### 2.1. Materials 

Seeds of the wheat cultivar Shiluan 02-1 were used in this study. Wheat flour and stone-milled whole wheat flour were provided by Sichuan Fenglaohan Technology Co., Ltd. (Jiangyou, China). To obtain whole wheat flour, the wheat was ground in a stone mill with a diameter of 600 mm and a grinding speed of 28–30 rpm, then passed through a 125 µm sieve. The contents of moisture, ash, fat, and protein in the flour were determined by AACC 44-01.01, 08-12.01, 30-25.01, and 46-12.01 [13], respectively. The wheat flour was 13.60% moisture, 0.55% ash, 1.12% fat, and 9.62% protein. The stone-milled whole wheat flour was 12.89% moisture, 1.42% ash, 1.60% fat, and 12.39% protein. Commercially dried whole wheat noodles (CDWWNs) were purchased from Xiangnian Food Co., Ltd. (Nanyang, China).

### 2.2. Preparation of the Dried Noodles

200 g of flour, 75 g of purified water, and 1.70 g of salt were mixed for 5 min. The prepared dough was placed in a sealed plastic bag and matured for 15 min at room temperature. Then, the dough sheet was pressed using a noodle-making machine (DMT-5, Longkou, China) until it was smooth. Then, the sheet was cut into fresh noodles of 1 mm thickness and 3 mm width. Following the drying at room temperature for 24 h, the dried noodles were cut into 22 cm length.

### 2.3. Cooking Quality

The water uptake ratio and cooking loss were determined by referring to the methods of Liu et al. [14] and Wang et al. [15], respectively. Dried noodles (10 g) with a W% moisture content were weighed (m_1_) and cooked in 500 mL boiling water for the optimum cooking time. Then, the cooked noodles were taken out and measured weight (m_2_) after absorbing the water, with two pieces of filter paper for 5 min. Then, the cooked noodles were collected and dried in an air oven at 135 °C until they reached a constant weight (m_3_). The water uptake ratio and cooking loss were calculated with the following Formulas (1) and (2), respectively. The noodle soup was cooled to room temperature, fixed to 500 mL with deionized water, and shaken well, and the turbidity of the noodle soup was measured at 720 nm.

(1) $$\mathrm{Water}\;\mathrm{uptake}\;\mathrm{ratio}\;(\%)=\frac{m_{2}-m_{1}}{m_{1}}\times 100$$

(2) $$\mathrm{Cooking}\;\mathrm{loss}\;(\%)=\frac{m_{1}\times (1-W)-m_{3}}{m_{1}\times (1-W)}\times 100$$

### 2.4. Texture Analysis

The texture properties were tested using a TA.XT plus texture analyzer (Stable Micro Systems, London, UK), according to Liu et al. [14], with some modifications. For the bending characteristics of the dried noodles, a length of 10 cm of dried noodles was taken, and the HDP/3PB probe was used. The testing parameters were: pre-test, 2 mm/s; test speed, 2 mm/s; post-test speed, 10 mm/s; distance, 15 mm. For the tensile properties, both ends of the cooked noodle were coiled on the A/PT probe, until the noodle was pulled off. The testing parameters were: pre-test, 1.67 mm/s; test speed, 3.33 mm/s; post-test speed, 10 mm/s. For the texture profile analysis (TPA), the dried noodles were cooked in 500 mL boiling water for the optimum cooking time, and cooled in cool water for 15 s, and filtered for 30 s. Three noodles were placed side by side on the carrier table, and the P36 R probe was used. The testing parameters were: pretest, test, and post-test speed, 0.2 mm/s; compression, 40%; interval time, 2 s.

### 2.5. Scanning Electron Microscopy (SEM)

The raw noodles were broken into small squares of 1~2 mm, fixed on the sample table with the broken side facing upward for spraying gold, and observed using a scanning electron microscope (EVO18, Zeiss, Oberkochen, Germany). The morphologies of the noodles were imaged with an accelerating voltage of 25 kV.

### 2.6. Fourier Transform Infrared Spectroscopy (FT-IR) 

The raw and cooked noodles were freeze-dried, then 1 mg of the tested sample and 100 mg KBr were put into an agate mortar, ground well, and pressed. The FTIR spectra were obtained by scanning from 400 to 4000 cm^−1^ with a resolution of 4 cm^−1^ [16]. The secondary structure of the protein was analyzed by deconvoluting the amide I region (1600–1700 cm^−1^). The spectra were analyzed using Omnic 8.2 and Peakfit 4.12 software for measuring the relative area of the corresponding amide I region [17]. The spectra were baseline-corrected and deconvoluted in the range of 800–1200 cm^−1^ with a peak width of 40 cm^−1^, and an enhancement factor of 1.9. The ratio of the peak intensity at 1047/1022 cm^−1^ reflected the ratio of the short-range ordered structure in starch [18].

### 2.7. In Vitro Antioxidant Activity

The method for extracting the phenolic substances from the raw and cooked noodles was obtained from a published study, with slight modifications [19]. The 2 g tested sample was placed in a centrifuge tube and 9 mL 80% ethanol was added. It was incubated at 50 °C for 30 min in an ultrasonic cleaner (RHCX-350, Jining, China) and centrifuged at 3200× *g* for 10 min. The obtained supernatant was collected and fixed to 10 mL with 80% ethanol. The DPPH radical scavenging capacity and hydroxyl radical scavenging capacity were measured, as described previously [20]. 

The ABTS radical scavenging activity was determined using the method followed by Sridhar et al. [21], with subtle adjustments. A 0.1 mL measurement of the sample solution was mixed with 4 mL of the ABTS^+^• working solution, the mixture reacted in the dark for 6 min, and the absorbance was measured at 734 nm. Deionized water was used instead of the sample solution as the blank group, and deionized water instead of the ABTS^+^• working solution as the control group. The ABTS^+^• scavenging activity was determined using the formula (3):

(3) $$\mathrm{ABTS}\;\mathrm{radical}\;\mathrm{scavenging}\;\mathrm{activity}\;(\%)=(1-\frac{A_{\mathrm{sample}}-A_{\mathrm{control}}}{A_{\mathrm{blank}}})\times 100$$

The total reducing power was measured using the method suggested by Zhang et al. [22], with a slight revision. A 0.5 mL sample, 2.5 mL of phosphate buffer (0.2 mol/L, pH 6.6), and 2.5 mL of 1% potassium ferricyanide were mixed, and the reaction mixture was incubated at 50 °C for 20 min in a water-bath (HH-S, Changzhou, China). Subsequently, 2.5 mL of 10% trichloracetic acid, 2.5 mL of deionized water, and 0.5 mL of 0.1% ferric chloride were added, and the mixture was allowed to stand for 10 min. Deionized water was substituted for the sample solution as the blank group, and the absorbance was recorded at 700 nm.

### 2.8. Antioxidant Activity after the In Vitro Simulated Digestion

The simulated saliva and gastric fluid were prepared, according to the method reported by Gawlik-Dziki et al. [23]. The simulated intestinal fluid was prepared by dissolving 6.80 g KH_2_PO_4_ in 500 mL of deionized water. The pH was adjusted to 6.8 with the addition of 0.1 mol/L NaOH. Finally, 0.20 g of trypsin was added to each 100 mL of buffer.

The in vitro digestion model was carried out with subtle adjustments [24]. In the oral digestion step, each 6 g of cooked noodles was mixed with 30 mL of simulated saliva. The mixture was stirred in a homogenizer (FSH-2A, Changzhou, China) and then shaken at 120× *g* for 2 min at 37 °C. In the gastric digestion step, the mixture of the remaining noodles from the last step and 30 mL of simulated gastric fluid was adjusted to pH 3.0, with the addition of 0.1 mol/L NaHCO_3_ and then shaken at 120× *g* for 2 h at 37 °C. In the intestinal digestion step, the mixture of the remaining noodles from the last step and 30 mL of the simulated intestinal fluid was adjusted to pH 7.0, with the addition of 0.1 mol/L NaOH and then shaken at 120× *g* for 3 h at 37 °C. The samples digested by mouth, stomach, and intestine were inactivated and then centrifuged at 3200× *g* for 10 min, respectively, and the supernatant was used to determine the antioxidant activity.

### 2.9. In Vitro Digestive Properties

The in vitro digestibility of the noodles was measured, according to the previous report, with a slight revision [25]. First, 0.5 g of freeze-dried cooked noodles was added to 10 mL of sodium acetate buffer (0.2 M, pH 6.0), and then l0 mL (300 U/mL) of α-amylase and 40 μL (100,000 U/mL) of glycolytic enzyme were added after thorough mixing. The mixture was shaken at 120× *g* in a constant temperature shaker (THZ-82A, Changzhou, China) at 37 °C, and 2 mL of the digest was taken at 0, 20, 30, 60, 90, 120, 150, and 180 min, respectively, and then centrifuged at 3200× *g* for 5 min, following 5 min of enzyme inactivation in a boiling water bath. Glucose in the supernatant was measured using the 3,5-dinitrosalicylic acid (DNS) method [26]. The contents of the rapidly digestible starch (RDS), the slowly digestible starch (SDS), and the resistant starch (RS) were calculated, according to Zhi et al. [27]. The starch hydrolysis rate was calculated, based on the method described elsewhere [28].

### 2.10. Estimated Glycemic Index (eGI)

The starch digestion curves were plotted using the starch hydrolysis rate as the vertical coordinate, and time as the horizontal coordinate. The curve followed the non-linear model established by Goni et al. [29]. The starch hydrolysis kinetics and area under curve (AUC) were calculated with the following Equations (4) and (5), respectively.

(4) $$C_{t}{=C}_{\infty }(1-e^{-\mathrm{kt}})$$

(5) $${\mathrm{AUC}=C}_{\infty }{(t}_{\infty }-t_{0})-(\frac{C_{\infty }}{k})[1-e^{-{k(t}_{\infty }-t_{0})}]\;$$

C_t_ is the percentage of digested starch at time t (min), C_∞_ is the equilibrium concentration of starch hydrolyzed after 180 min, k is the kinetic constant, t_∞_ is the final time point (180 min), and t_0_ is the initial time point (0 min). 

The hydrolysis index (HI) was obtained by dividing the area under the hydrolysis curve of the noodles by the corresponding area of the reference sample (white bread). The eGI was calculated, according to the HI value by Formula (6).

(6) eGI=0.862HI+8.198

### 2.11. Statistical Analysis

The mean values with standard deviations of the replicate measurements are presented. The statistical analysis was performed using SPSS 21.0 (IBM, Chicago, IL, USA). The images were drawn using Origin 2021 (Stat-Ease Inc., Minneapolis, MN, USA).

## 3. Results and Discussion

### 3.1. Cooking Quality

As shown in Table 1, the water uptake ratio and cooking loss of the three kinds of dried noodles were significantly different (*p* < 0.05). Wheat gluten protein had a good water absorption, whereas bran weakened the three-dimensional structure formed by the gluten protein, to a certain extent, reduced the water holding capacity of the gluten protein, and reduced the water uptake ratio of the whole wheat noodles [30]. The noodle soup of CDWWNs was yellowish brown, and the turbidity of the noodle soup was significantly higher than that of the DWNs and SDWWNs (*p* < 0.05), which could be ascribed to the precipitation of amylose, due to the disruption of the gluten matrix continuity [12]. In addition, Sim et al. [9] found that the solubility and hydration capacity of bran may affect the cooking loss of the noodles.

### 3.2. Texture Properties

The greater the breaking force and breaking distance of the raw dried noodles, the more flexible they are and less likely to break during packaging, transportation, and sales. As can be seen from Table 2, the difference in the breaking force of the three raw noodles was significant (*p* < 0.05), and the breaking force of the wheat noodles was much less than that of the two whole wheat noodles, which was only 0.73 N. The breaking force of the raw noodles is related to the protein content of the flour, while whole wheat flour contains all of the proteins of wheat seeds, resulting in a highly stable starch-protein-fat complex [31]. The better the tensile properties of the cooked noodles, the more elastic they are. The tensile strength, extensibility, and elongation of the SDWWNs were higher than those of the DWNs and CDWWNs. On the one hand, the high water-holding capacity of the dietary fiber is conducive to maintaining the gluten network structure and increasing the tensile strength of the noodles; on the other hand, the cross-linking of pentosan, ferulic acid, proteins, and starch improves the tensile properties of the noodles [32]. The formation of hydrogen bonds between the hydroxyl groups of starch molecules and water molecules and the covalent cross-linking of the proteins may be the main factors determining the extensibility of the cooked noodles [33].

In Table 3, the hardness of the SDWWNs and CDWWNs was significantly higher than that of the DWNs (*p* < 0.05), probably because the bran supported the internal structure of the noodles and formed a stable structural system, resulting in an increased hardness [30]. The springiness, cohesiveness, gumminess, chewiness, and resilience of the SDWWNs reached the maximum value, which may be attributed to the pre-pasting of starch caused by the stone mill, which helps amylose form a three-dimensional network interconnected by microcrystalline bundles and improves the texture properties of the noodles [34]. The springiness of the noodles may be related to the high amylose content, which deteriorates to form a gel during the cooling of the noodles, resulting in an increased springiness. In addition, the dietary fiber may fill in the gluten structure, increase the strength of the network structure, and result in an increased hardness, gumminess, and chewiness of the noodle, because it has a strong water-holding capacity and could be bound to proteins through the oxidative cross-linking of the active double bonds [35]. 

### 3.3. Microstructure

**Figure 1:**
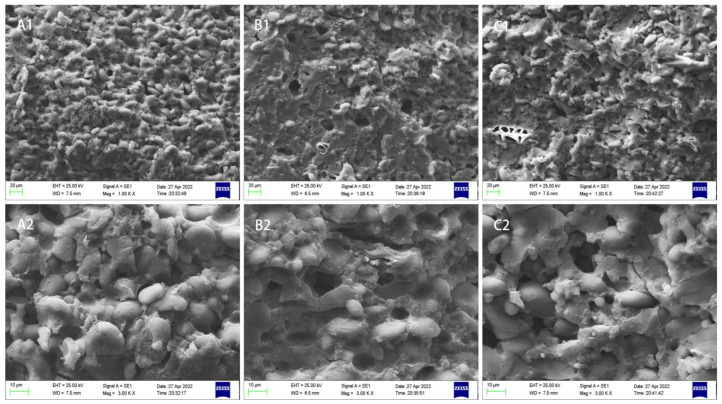
Inner microstructure of the raw noodles: (**A**) dried wheat noodle; (**B**) stone-milled dried whole wheat noodle; (**C**) commercially dried whole wheat noodle. Figures numbered 1 and 2 were taken at 1000× and 3000× magnification, respectively.

As shown in Figure 1, the microstructure of the raw noodles was mainly composed of the gluten protein reticulation structure, and the larger disc-shaped A-type starch, and the smaller oblate spherical B-type starch [30]. The microstructure of the DWNs was continuous and dense, with a uniform gluten network structure and large starch granules. Compared with the DWNs, the gluten network continuity of the SDWWNs and CDWWNs was reduced, and more holes appeared as a result of the presence of bran, which prevented gliadin and glutenin from linking with each other through disulfide bonds and forming an orderly mesh structure [36]. The number and area of holes in the SDWWNs were significantly increased, probably because the whole wheat flour obtained by crushing wheat seeds in the stone mill was not uniformly distributed and because of the large grain size of the individual bran, which was not fully integrated with the gluten network [3].

### 3.4. Analysis of the FT-IR

**Figure 2:**
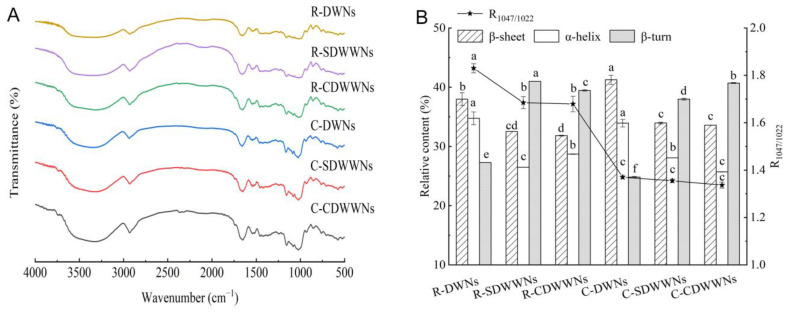
**(A**) FT-IR spectra of the raw and cooked noodles; (**B**) The changes of the secondary structure of proteins and short-range ordered structure of the raw and cooked noodles. DWNs, dried wheat noodles; SDWWNs, stone-milled dried whole wheat noodles; CDWWNs, commercially dried whole wheat noodles. Different letters indicate significant differences in the same indicator data (*p* < 0.05).

Figure 2A shows that the FT-IR spectra of the raw and cooked noodles were basically similar, and the peak shapes and positions of the characteristic peaks did not change significantly. The intensity of the absorption peak of the noodles changed after cooking, probably due to changes in the content of the functional groups or in the mode of combining. The secondary structure of proteins includes the β-sheet structure (1600–1640 cm^−1^), random coil structure (1640–1650 cm^−1^), α-helix structure (1650–1660 cm^−1^), and β-turn structure (1660–1700 cm^−1^) [37]. The protein secondary structure and R_1047/1022_ of the noodles are shown in Figure 2B. The protein secondary structure of the SDWWNs and CDWWNs was mainly composed of the β-sheet and β-turn. The β-sheet was regarded as the most stable protein conformation, and the α-helix was also a relatively stable conformation [38]. It is possible that the high dietary fiber content reduced the continuity of the gluten protein network structure and weakened the hydrogen bonding between the protein polypeptide chains, leading to an increase in the disordered structures within the protein molecules [39]. The increased β-sheet content of the cooked noodles may be explained by the fact that the high temperature changed the structure of the protein molecules from curled to stretched, and the protein aggregation was promoted by the interaction between the protein molecules [40]. Compared with the raw noodles, the three cooked noodles R_1047/1022_ were significantly decreased (*p* < 0.05), probably because the starch was hydrolyzed after absorbing water and swelling during cooking, and the hydrogen bonds between the molecular chains were broken and the helical structure was unraveled [30].

### 3.5. In Vitro Antioxidant Activity

**Figure 3:**
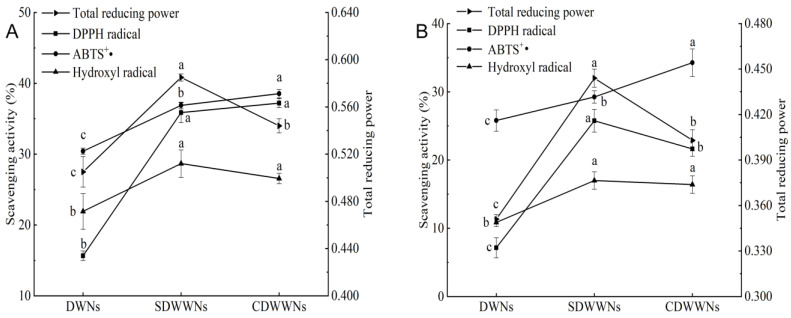
In vitro antioxidant activity of the raw and cooked noodles. (**A**) raw noodles; (**B**) cooked noodles. DWNs, dried wheat noodles; SDWWNs, stone-milled dried whole wheat noodles; CDWWNs, commercially dried whole wheat noodles. Different letters indicate significant differences in the same indicator data (*p* < 0.05).

The antioxidant activity of the whole wheat noodles was higher than that of the wheat noodles, due to the presence of carotenoids, polyphenols, alkylresorcinols, phytic acid, and other antioxidant components in the bran and germ [1]. In Figure 3, the differences in the DPPH radical scavenging activity, the ABTS^+^• scavenging activity, and the total reducing power of the three cooked dried noodles were significant (*p* < 0.05). The DPPH radical scavenging activity, hydroxyl radical scavenging activity, and total reducing power of the cooked-SDWWNs were the highest, which were 25.76%, 17%, and 0.444, respectively. The antioxidant activity of the cooked noodles was reduced, compared to the raw noodles, probably because the phenolic substances in the noodles were destroyed during high-temperature cooking and lost to the noodle soup.

### 3.6. Antioxidant Activity after the In Vitro Simulated Digestion

**Figure 4:**
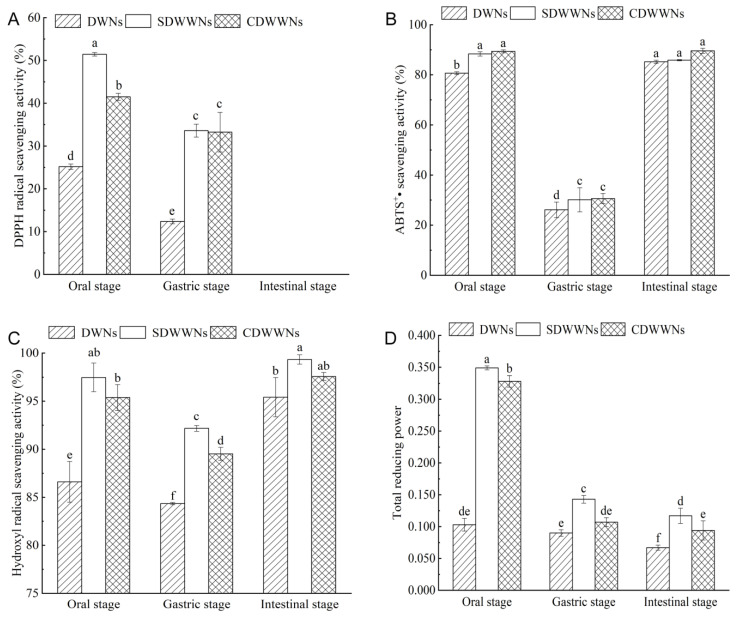
Antioxidant activity of the cooked noodles after the in vitro simulated digestion. (**A**) DPPH radical scavenging activity; (**B**) ABTS radical scavenging activity; (**C**) Hydroxyl radical scavenging activity; (**D**) Total reducing power. DWNs, dried wheat noodles; SDWWNs, stone-milled dried whole wheat noodles; CDWWNs, commercially dried whole wheat noodles. Different letters indicate significant differences in the same indicator data (*p* < 0.05).

In the oral digestion stage, the DPPH radical scavenging activity, the hydroxyl radical scavenging activity, and the total reducing power were in the order of SDWWNs > CDWWNs > DWNs (Figure 4). Compared with the simulated oral digestion stage, the antioxidant activity of the noodles was reduced to different degrees after the gastric digestion, probably due to the release of some phenolic compounds by the oral amylase action [41]. In addition, on the one hand, pepsin promoted the degradation or conversion of the antioxidant active ingredients under acidic conditions, such as the loss of some water-soluble polyphenols; on the other hand, the phenolic compounds combined with pepsin, such that the antioxidant activity was reduced [42]. The noodles no longer had the ability to scavenge the DPPH radical after the intestinal digestion, which may be owed to the excessive concentration of the phosphate buffer, resulting in the deepening of the color of the DPPH radical [43]. Compared with the gastric digestion phase, the ABTS^+^• scavenging activity and hydroxyl radical scavenging activity of the noodles were elevated after the intestinal digestion, probably on account of the increase in pH leading to the improvement of the stability of the phenolic compounds [44].

### 3.7. In Vitro Digestive Properties of the Noodles

**Figure 5:**
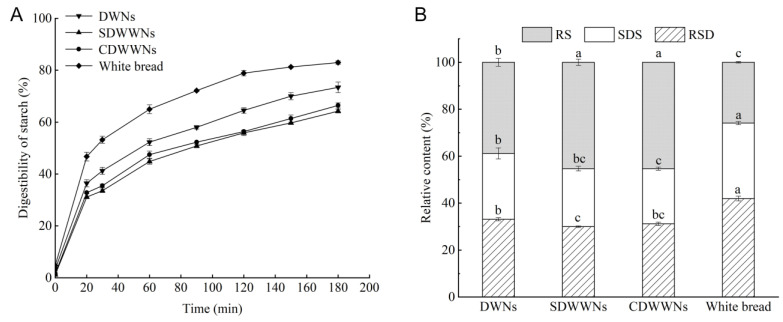
(**A**) Digestion progress curves of the cooked noodles; (**B**) The contents of the RDS, SDS, and RS of the cooked noodles. DWNs, dried wheat noodles; SDWWNs, stone-milled dried whole wheat noodles; CDWWNs, commercially dried whole wheat noodles. Different letters indicate significant differences in the same indicator data (*p* < 0.05).

As shown in Figure 5, the RDS, SDS, and RS contents of white bread were significantly different from those of the noodles (*p* < 0.05), probably because white bread was loose and porous, which increased the contact area with glycosylase and α-amylase and promoted the starch digestion and absorption [45]. Compared with wheat noodles, the cooking loss rate of whole wheat noodles was higher, and the soluble starch was dissolved, leading to a reduction in the digestible starch content. The RS content of the wheat noodles was significantly lower (*p* < 0.05) than that of the whole wheat noodles, mainly because the whole wheat noodles are rich in dietary fiber and resistant starch. The differences between the RDS, SDS, and RS contents of the SDWWNs and CDWWNs, were not significant (*p* > 0.05), probably because part of the whole wheat flour had been gelatinized after the stone milling and the starch structure had been destroyed, which promoted the decomposition of the starch by the digestive enzymes. Furthermore, the rich protein in the stone-milled whole wheat flour was tightly bound to the starch to prevent the digestive enzymes from entering the starch granules [46].

It can be seen from Figure 5 that the starch hydrolysis rate of all samples increased rapidly during the first 20 min of digestion and slowly increased from 20 to 180 min. At the end of the digestion, the starch hydrolysis rate was in the order of white bread (82.96%) > DWNs (73.36%) > CDWWNs (66.41%) > SDWWNs (64.25%). On the one hand, the soluble dietary fiber in the whole wheat noodles is cross-mixed with starch, which hinders the interaction of glycosylase and α-amylase with starch, to a certain extent; on the other hand, it dissolves in water to form a gel-like semi-fluid, reducing the hydrolysis rate of α-amylase [47]. Additionally, the polyphenols not only form polyphenol-starch complexes with starch, but also bind to the digestive enzymes, resulting in a lower glycosylase and α-amylase activity [48].

### 3.8. Estimated Glycemic Index (eGI)

In Table 4, the C_∞_, AUC, HI, and eGI of the DWNs, were significantly higher than those of the SDWWNs and CDWWNs (*p* < 0.05). The higher the RS content, the lower the C_∞_ and eGI. Different samples were different in k. The difference between k for the noodles and white bread was significant (*p* < 0.05), with smaller values indicating a slower in vitro digestion of starch to C_∞_. The eGI value was in the order of white bread (94.4) > DWNs (79.23) > CDWWNs (71.51) > SDWWNs (69.37). The eGI of different dried noodles was significantly lower than that of white bread (*p* < 0.05), which indicates that dried noodles are a good carrier for processing low eGI foods. The eGI value of SDWWNs was significantly lower than that of CDWWNs (*p* < 0.05), which was probably caused by the higher dietary fiber content and proportion of amylase to amylopectin of SDWWNs, resulting in the differences in the in vitro digestion properties of starch [49]. Moreover, previous studies recommended that increasing the intake of whole wheat food has a valuable effect on human health because of the high content of dietary fiber, phenolic compounds, non-starch polysaccharides, and other nutritive and functional components [1,2]. In Asia, wheat noodles are an important staple food, but they can cause a rapid increase in blood sugar. Therefore, SDWWNs may be used as a staple food for special groups, such as diabetic patients, because it can properly regulate the level of the blood sugar response, and prevent and control cardiovascular and cerebrovascular diseases [50].

## 4. Conclusions

The SDWWNs performed best, in terms of the water uptake ratio (154.68%), cooking loss (6.31%), turbidity of the noodle soup (0.085), and tensile strength (0.24 N). The gluten network continuity of the SDWWNs and CDWWNs was reduced, compared to the DWNs, and more holes appeared, as a result of the presence of bran. The number and area of holes in the SDWWNs were also significantly increased. The protein secondary structure of the SDWWNs and CDWWNs was mainly composed of the β-sheet and β-turn. The high temperature changed the curled to stretched structure of the protein molecules, and the protein aggregation was promoted by the interaction between the protein molecules, so the β-sheet content of the cooked noodles increased. Prior to and after the in vitro simulated digestion, the DPPH radical scavenging activity, the hydroxyl radical scavenging activity, and the total reducing power of the SDWWNs were the highest. The RDS, SDS, and RS contents of the three kinds of noodles were different, and the starch hydrolysis rate was in the order of DWNs (73.36%) > CDWWNs (66.41%) > SDWWNs (64.25%). At the same time, the eGI value of the SDWWNs was the lowest. Overall, the SDWWNs had obvious advantages, in terms of cooking quality, textural properties, antioxidant activity, and controlling the glycemic response.

## Figures and Tables

**Table 1:** Cooking quality of three kinds of dried noodles.

Sample	Water Uptake Ratio (%)	Cooking Loss (%)	Turbidity of the Noodle Soup
DWNs	165.58 ± 1.87 ^a^	3.98 ± 0.16 ^c^	0.072 ± 0.011 ^b^
SDWWNs	154.68 ± 2.26 ^b^	6.31 ± 0.11 ^b^	0.085 ± 0.004 ^b^
CDWWNs	140.52 ± 3.04 ^c^	7.05 ± 0.08 ^a^	0.111 ± 0.005 ^a^

DWNs, dried wheat noodles; SDWWNs, stone-milled dried whole wheat noodles; CDWWNs, commercially dried whole wheat noodles. Different letters indicate significant differences in the same indicator data (*p* < 0.05).

**Table 2:** Bending properties of the raw noodles and the tensile properties of the cooked noodles.

Sample	Breaking Force (N)	Breaking Distance (mm)	Tensile Strength (N)	Extensibility (mm)	Elongation (%)
DWNs	0.73 ± 0.05 ^c^	4.21 ± 0.25 ^b^	0.16 ± 0.01 ^c^	24.66 ± 4.29 ^a^	87.77 ± 14.67 ^a^
SDWWNs	0.90 ± 0.06 ^b^	4.29 ± 0.18 ^ab^	0.24 ± 0.02 ^a^	28.30 ± 5.36 ^a^	105.92 ± 22.41 ^a^
CDWWNs	0.97 ± 0.08 ^a^	4.41 ± 0.23 ^a^	0.21 ± 0.02 ^b^	25.72 ± 3.35 ^a^	92.53 ± 11.34 ^a^

DWNs, dried wheat noodles; SDWWNs, stone-milled dried whole wheat noodles; CDWWNs, commercially dried whole wheat noodles. Different letters indicate significant differences in the same indicator data (*p* < 0.05).

**Table 3:** Total texture properties of three kinds of dried noodles.

Sample	Hardness (N)	Springiness	Cohesiveness	Gumminess (N)	Chewiness (N)	Resilience
DWNs	14.85 ± 0.74 ^b^	0.89 ± 0.02 ^ab^	0.76 ± 0.02 ^c^	11.28 ± 0.66 ^c^	9.98 ± 0.52 ^c^	0.36 ± 0.03 ^c^
SDWWNs	21.79 ± 0.84 ^a^	0.91 ± 0.04 ^a^	0.81 ± 0.02 ^a^	17.67 ± 0.79 ^a^	16.11 ± 1.20 ^a^	0.49 ± 0.02 ^a^
CDWWNs	21.91 ± 0.98 ^a^	0.87 ± 0.05 ^b^	0.78 ± 0.02 ^b^	17.00 ± 0.70 ^b^	14.88 ± 1.23 ^b^	0.43 ± 0.02 ^b^

The springiness, cohesiveness, and resilience are dimensionless. DWNs, dried wheat noodles; SDWWNs, stone-milled dried whole wheat noodles; CDWWNs, commercially dried whole wheat noodles. Different letters indicate significant differences in the same indicator data (*p* < 0.05).

**Table 4:** Digest model parameters and estimated glycemic index of the dried noodles.

Sample	C_∞_ (%)	k (min^−1^)	AUC	HI (%)	eGI
White bread	80.23 ± 0.66 ^a^	0.033 ± 0.002 ^a^	12,042.91 ± 18.74 ^a^	100.00 ± 0.00 ^a^	94.40 ± 0.00 ^a^
DWNs	69.68 ± 1.51 ^b^	0.027 ± 0.003 ^b^	9923.35 ± 41.64 ^b^	82.40 ± 0.35 ^b^	79.23 ± 0.30 ^b^
SDWWNs	60.76 ± 0.38 ^c^	0.025 ± 0.001 ^b^	8546.87 ± 73.02 ^d^	70.97 ± 0.61 ^d^	69.37 ± 0.52 ^d^
CDWWNs	61.80 ± 1.21 ^c^	0.027 ± 0.001 ^b^	8845.60 ± 82.84 ^c^	73.45 ± 0.69 ^c^	71.51 ± 0.59 ^c^

C_∞_, the equilibrium concentration of starch hydrolyzed after 180 min; k, the kinetic constant; AUC, area under curve; HI, hydrolysis index; eGI, estimated glycemic index; DWNs, dried wheat noodles; SDWWNs, stone-milled dried whole wheat noodles; CDWWNs, commercially dried whole wheat noodles. Different letters indicate significant differences in the same indicator data (*p* < 0.05).

## Data Availability

Data is contained within the article.
